# Supplementary material for: Refining animal care through technology: Addressing alopecia in Jaculus jaculus with validated computer vision analysis
Source: PLoS One. 2025 Nov 11;20(11):e0330143. doi: 10.1371/journal.pone.0330143 (PMC12604758; doi:10.1371/journal.pone.0330143)
Supplement: S1 Table — (DOCX) [file pone.0330143.s001.docx]

**S1 Table:** Mutually exclusive ethogram for captive singly housed jerboa during the dark photoperiod utilized for observer rating.

| **Title** | **Description** |
| --- | --- |
| Obstructed | Unable to visualize; may involve nesting behaviors or behind the provided dust bath. |
| Jumping | Both feet leave contact with ground while upright, typically involving vertical movement. |
| Upright, Rapid Movements | Animal is upright, nonambulatory or single stepping and not interacting with the food bowl. Small, rapid movements of the hands may be observed. May involve grooming of the muzzle or hands, foraging, or prehension of food. |
| Food Bowl Interactions | Upright and interacting with crock that provides food with both feet in contact with the ground or crock, may involve sitting in or perching on the rim of the crock. |
| Ambulating | Movement resulting in translocation with an upright animal and at least one foot in contact with ground. |
| Grooming | Movement of forelimbs, feet, or muzzle to contact the body, limbs, or tail. This may involve prehension of the tail to present to the muzzle or licking, biting, or chewing fur on the body. Please note that this does not include presentation of the hands to the muzzle specifically. |
| Wall Interactions | Animal is upright, located and directed towards the wall of the enclosure. At least one foot is in contact with ground, legs may be in full extension, and may involve repetitive clawing or digging motions against the wall. |
| Dust Bath Interactions | Visualized within the dust bath, may include entering, exiting, rolling, interacting with the walls of, and being sedentary within the dust bath. |
| Rolling | Transitioning to or in lateral recumbency, and potentially involving successive flexion and extension of the legs. |
