# Supplementary material for: Refining animal care through technology: Addressing alopecia in Jaculus jaculus with validated computer vision analysis
Source: PLoS One. 2025 Nov 11;20(11):e0330143. doi: 10.1371/journal.pone.0330143 (PMC12604758; doi:10.1371/journal.pone.0330143)
Supplement: S2 Table — (DOCX) [file pone.0330143.s002.docx]

**S2 Table:** Summary of individual jerboa enrichment item introduction schedule. ‘X’ represents data collection. A one-week washout period was utilized between items. All animals were additionally recorded the day before item introduction. Items were introduced in a randomized order, and jerboa were recorded for an hour during the dark photoperiod.

|  |  | Wood Stick | | | Tunnel | | | Divider | | |
| --- | --- | --- | --- | --- | --- | --- | --- | --- | --- | --- |
| Animal Number | Day: | 1 | 7 | 14 | 1 | 7 | 14 | 1 | 7 | 14 |
| I |  | X | X | X | X | X | X | X | X | X |
| II |  | X | X | X | X | X | X | X | X | X |
| III |  | X | X | X | X | X | X | X | X | X |
| IV |  | X | X | X | X | X | X | X | X | X |
| V |  | X | X | X | X | X | X | X | X | X |
| VI |  | X |  |  |  |  |  |  |  |  |
| VII |  |  |  |  |  |  |  | X |  |  |
| VIII |  | X |  |  |  |  |  |  |  |  |
