# Supplementary material for: Refining animal care through technology: Addressing alopecia in Jaculus jaculus with validated computer vision analysis
Source: PLoS One. 2025 Nov 11;20(11):e0330143. doi: 10.1371/journal.pone.0330143 (PMC12604758; doi:10.1371/journal.pone.0330143)
Supplement: S3 Table — (DOCX) [file pone.0330143.s003.docx]

**S3 Table:** Description of additional exclusive ethogram terms with the addition of enrichment items.

| **Title** | **Description** |
| --- | --- |
| Wood Item Interaction | Interacting with the provided wooden enrichment item, may include moving, prehending, standing on, or chewing. |
| Tunnel Interaction | Interacting with the provided tunnel enrichment item, may include moving, prehending, packing with substrate or nest material, standing on or within, chewing, or rolling. |
