# Supplementary material for: Refining animal care through technology: Addressing alopecia in Jaculus jaculus with validated computer vision analysis
Source: PLoS One. 2025 Nov 11;20(11):e0330143. doi: 10.1371/journal.pone.0330143 (PMC12604758; doi:10.1371/journal.pone.0330143)
Supplement: S4 Table — (DOCX) [file pone.0330143.s004.docx]

**S4 Table:** Summary of enrolled animals. Video data collected utilized to generate the following: 1) Exhaustive Ethograms, 2) Exclusive Ethogram (Supplemental Table 1), 3) Activity Budget (data in Fig. 1), 4) Comparison Subset, and 5) Enrichment Assessment (see Supplemental Table 3). N.B. Animal II was moved between activity budget and enrichment recording.

| Animal ID | Sex | Age at Recording (months) | Cage Height (cm) | Position on Rack | Video Utility |
| --- | --- | --- | --- | --- | --- |
| I | Female | 33 | ~35 | Middle | 1, 2, 3, 4, 5 |
| II | Male | 9 | ~35 | Top | 5 |
| II | Male | 8 | ~51 | Middle | 2, 3 |
| III | Male | 12 | ~35 | Bottom | 2, 3, 4, 5 |
| IV | Male | 17 | ~51 | Bottom | 1, 4, 5 |
| V | Female | 17 | ~35 | Middle | 2, 3, 5 |
| VI | Male | 3 | ~35 | Top | 2, 3, 5 |
| VII | Male | 20 | ~51 | Bottom | 2, 3, 5 |
| VIII | Male | 7 | ~51 | Middle | 2, 3, 4, 5 |
| IX | Female | 34 | ~33 | Bottom | 2, 3 |
| X | Male | 27 | ~51 | Middle | 2, 3, 4 |
| XI | Female | 27 | ~35 | Top | 2, 3 |
| XII | Female | 3 | ~35 | Middle | 2, 3 |
| XIII | Male | 26 | ~35 | Middle | 4 |
